# Effects of Different Non-Cage Housing Systems on the Production Performance, Serum Parameters and Intestinal Morphology of Laying Hens

**DOI:** 10.3390/ani11061673

**Published:** 2021-06-04

**Authors:** Yi Wan, Huan Yang, Hongyi Zhang, Ruiyu Ma, Renrong Qi, Junying Li, Wei Liu, Yan Li, Kai Zhan

**Affiliations:** 1 Anhui Key Laboratory of Livestock and Poultry Product Safety Engineering, Institute of Animal Husbandry and Veterinary Medicine, Anhui Academy of Agriculture Science, No. 40 Nongke West Road, Hefei 230031, China; eternalwan@163.com (Y.W.); 18655160470@163.com (R.M.); easona123@126.com (R.Q.); brandon2007@163.com (J.L.); liuweicau@163.com (W.L.); Liyan1314526@126.com (Y.L.); 2 College of Animal Science and Technology, Anhui Agricultural University, Hefei 230001, China; 18855031103@163.com; 3 Hubei Shendan Health Food Co., Ltd., Anlu 432600, China; zhyzhyyhz2@sina.com

**Keywords:** laying hen, non-cage housing system, production performance, serum parameter, intestinal morphology

## Abstract

**Simple Summary:**

With the increased interest in animal welfare, poultry housing systems have been a concern for the last decade, and conventional cages have been replaced by non-cage systems or enriched cages. However, the environmental conditions in non-cage housing systems and their association with hens’ production performance and health have not been studied extensively. Therefore, the present study compared the indoor environmental microbial content in two different non-caged systems, namely, a plastic-net housing system and floor-litter housing system, and investigated its effects on the production performance, serum parameters and intestinal morphology of hens during the peak laying period. The results indicated that the NRS resulted in better indoor environmental air quality and ground hygiene than the LRS and enhanced the production performance, antioxidant capacity and intestinal health of hens, represented by positive changes in the laying rate, serum parameters and intestinal morphology.

**Abstract:**

This study investigated the effects of plastic-net housing system (NRS) and floor-litter housing system (LRS) on the production performance, serum parameters and intestinal morphology of Shendan laying hens. A total of 1200 30-week-old hens were randomly allocated to the NRS and LRS groups, each of which included five replicates with 120 chickens in each replicate. The experiment was conducted from 32 to 40 weeks of age. Indoor airborne parameters were measured every 2 weeks, and indoor ground contamination was measured monthly. The laying rate and mortality of hens were recorded daily, and egg quality traits and serum parameters were measured every 2 weeks. At 40 weeks of age, four birds per replicate from each experimental group were selected for intestinal morphological observation. The results showed that the airborne bacteria number in the LRS was significantly higher than that in the NRS (*p* < 0.05) for most of the experimental period (except at 32 and 38 weeks of age), and the bacterial numbers on the surfaces of the floor and floor eggs in the LRS were approximately 10 times higher than those in the NRS (*p* < 0.05). Compared with the LRS, the NRS improved the laying rate (*p* < 0.05), reduced serum malondialdehyde (MDA) (*p* < 0.05) and corticosterone (CORT) concentrations and increased serum glutathione peroxidase (GSH-Px) and superoxide dismutase (SOD) activities, indicating favourable effects on antioxidative status. The NRS was significantly associated with an increased villus height (VH), villus height to crypt depth ratio (VCR) in the small intestine (*p* < 0.05) and increased VCR in the caecum (*p* < 0.05). Overall, the lower rate of bacterial contamination in the NRS than in the LRS indicated better environmental hygiene. The NRS enhanced the laying performance and antioxidant capacity of hens and was superior to the LRS in improving intestinal health. The current findings support the advantages of the NRS for the health and welfare of Shendan chickens during the peak laying period.

## 1. Introduction

The housing system is one of the most important non-genetic factors for hens that affects both production performance and health status [1,2]. There are several different housing systems in poultry production, mainly including the cage housing system (CRS), floor-litter housing system (LRS) and plastic-net housing system (NRS). In some countries, especially in North-West Europe, laying hens have been kept in non-cage systems for many years, as it is perceived as being more respectful to animal welfare than cage housing systems which could allow behavioural freedom and promote eco-friendliness [3,4]. The ban on housing hens in conventional cages has led to a search for more suitable non-cage housing systems.

Several studies have been conducted to evaluate the effects of NRS and LRS on poultry production; however, the results are not consistent. Almeida et al., (2017) [5] found that birds reared in the NRS had a higher laying rate than birds reared in the LRS. Zhang et al., (2018) [6] reported that the NRS decreased the high-density lipoprotein cholesterol content, and enhanced total protein and triacylglycerol contents of birds compared to the LRS. In contrast, Li et al., (2016) [7] favoured the LRS because it was associated with higher body weight gain and a greater number of Bifidobacteria in the caeca of 28-day-old broilers compared to the NRS. Wang et al., (2015) [8] concluded that broilers raised in the LRS had increased gizzard weights at days 21 and 42 compared to those raised in the NRS. However, the environmental conditions in non-cage housing systems and their association with laying hens’ hygiene and health have not been studied extensively. Aerosol microbial contamination was found to be higher in LRS than in the NRS [9]. Birds raised in NRS showed better hygiene and had a lower incidence of hock injury and footpad dermatitis than those reared in LRS [5]. Birds in aviaries with wire mesh flooring had fewer wounds on their body surface and lower mortality as compared to hens in aviaries with plastic-slatted flooring [10]. Although non-cage systems provide more opportunities to perform natural behaviours compared to cage systems, their environmental monitoring need to be further concerned.

In the present study, we hypothesised that the different non-cage systems might have great effects on hens due to their different circumstances and rearing methods. Therefore, two different non-cage housing systems, i.e., NRS and LRS, were used under the same management conditions to compare their indoor environmental microbial content and investigate their effects on the production performance, serum parameters and intestinal morphology of hens during the peak laying period.

## 2. Materials and Methods

The experimental protocol of the current study was approved by the Committee for the Care and Use of Experimental Animals at Anhui Academy of Agricultural Science under permit No. A11-CS06.

### 2.1. Animals and Management

The Shendan chicken, which originated in North China, is used as a dual-purpose breed and is one of the most well-known and popular local chicken breeds in Hubei Province. A total of 1200 30-week-old healthy, commercial Shendan laying hens with similar body weights (1295.20 g ± 106.54) and that were raised in cages were obtained from Hubei Shendan Health Food Co., Ltd., Anlu, China, and were randomly divided into the NRS and LRS groups. Each group included 5 replicate pens with 120 birds in each replicate. Birds in the NRS treatment group were raised indoors on a perforated plastic floor; the faeces dropped onto the belt under the plastic floor and were removed every day. Birds in the LRS treatment group were raised indoors on a floor covered with wood shavings that was cleaned every 2 weeks. Each replicate pen in both groups had the same indoor stocking density (4.4 birds/m^2^) and had a free-range area measuring 8 × 6 m (2.5 birds/m^2^). There was a plurality of nest boxes in indoor houses for hens to lay eggs. The free-range area, which was used as an activity field, was separated from surrounding areas by wire fences. Feeders and bell drinkers were located in both the indoor and free-range areas. There were also some yellow wooden perches available for the chickens to rest upon. A preliminary trial was conducted for 2 weeks, and the formal experiment was performed from week 32 to 40. The poultry houses with the two housing systems were close to one another.

### 2.2. Measurement of Indoor Airborne Parameters

The airborne bacteria obtained at different sites in each replicate pen (one plate for each front, middle and back side) were evaluated by the sedimentation plate method every 2 weeks. An uncovered culture plate (9 cm in diameter) containing 20 mL of culture medium (NA, Nutrient Agar) was distributed and exposed to air at 0.5 m above ground for 5 min. Thereafter, the plates were collected and were then incubated in the dark at 37 °C for 48 h using the thermostatic incubator (DRP-9052, Nade Scientific Instrument Co., Ltd., Zhejiang, China), and the airborne bacteria number was calculated by the following equation:

C = 50,000N/AT

where N is the number of colony-forming units (CFUs) per plate; A is the base area of the plate in cm^2^; T is the exposure time in min and C is the airborne bacteria number, CFU/m^3^. The airborne bacteria number for each replicate was the mean value of the front, middle and back sides. Simultaneously, the indoor temperature, relative humidity and CO_2_ concentration were recorded. The temperature and relative humidity were measured by the Portable Temperature Measuring Instrument (Fluke 971, Tianchuang Instrument Co., Ltd., Zhuhai, China). The CO_2_ concentration was detected by the Carbon Dioxide Gas Detector (GT-903-CO_2_, Korno Electronic Technology Co., Ltd., Shenzhen, China).

### 2.3. Measurement of Indoor Ground Contamination

#### 2.3.1. Sample Collection

To determine indoor ground bacterial contamination, three samples from three sites (from the front, middle and back sides) for the surface of the indoor floor, nest eggs and floor eggs, were collected. The surfaces were sampled with sterile swabs, which were then transferred into screw-cap tubes with 50 mL of DEPC (diethylpyrocarbonate) water. Samples were stored at −4 °C until analysis. All samples were collected every 4 weeks.

#### 2.3.2. DNA Extraction and PCR Amplification of 16S rRNA Sequences

The collected sample was filtered through a 0.22-µm membrane and eluted with 1 mL of DEPC water. Bacterial genomic DNA was extracted using a PowerMag Microbiome DNA isolation kit (OMEGA Bio-Tek, Norcross, GA, USA) according to the manufacturer’s instructions. The V4-V5 hypervariable regions of 16S rRNA genes were PCR-amplified from microbial genomic DNA using the universal primers V515F (5′-GTGCCAGCMGCCGCGGTAA-3′) and V907R (5′-CCGTCAATTCMTTTRAGTTT-3′). PCR was performed in a 20-μL reaction system containing 0.8 μL of each primer, 10 ng of template DNA, 4 μL of 5× FastPfu buffer, 2 μL of 2.5 mM dNTPs and 0.4 μL of FastPfu polymerase. The thermocycling parameters were as follows: a 2 min initial denaturation at 95 °C; 30 cycles of denaturation at 95 °C for 30 s, annealing at 50 °C for 30 s, and elongation at 72 °C for 45 s and a final extension at 72 °C for 10 min. The amplicons were pooled, purified and then quantified using a NanoDrop 2000 UV-vis instrument (Thermo Scientific, Wilmington, DE, USA).

#### 2.3.3. Preparation of the Plasmid Standard

The plasmid standard for quantitative real-time PCR (qRT-PCR) was constructed as follows. According to the amplification positions of the bacterial 16S rDNA universal primers (515-F/907-R), a 412-bp sequence, from *Escherichia coli* 16S rDNA position 515–926, was inserted into the cloning vector pUC57 (the plasmid standard in this experiment was obtained by gene synthesis), and the resulting recombinant standard plasmid was named pUC57-16S rDNA. The total length of pUC57-16S rDNA was 3122 bp, and the extracted plasmid concentration was 142.93 ng/µL. The copy number of pUC57-16S rDNA was 4.17 × 10^10^ copies/µL. The plasmid standard was diluted to 4.17 × 10^9^–4.17 × 10^3^ copies/µL with double-distilled water, and 7 concentrations were used to establish the standard curve for qRT-PCR.

#### 2.3.4. qRT-PCR

qRT-PCR (absolute quantification) was used to determine the bacterial 16S rDNA copy numbers in the samples. qRT-PCR was performed in a final reaction volume of 20 µL, containing 0.5 µL of each primer, 1 µL of template DNA, 10 µL of 2× TB Green Premix Ex Taq II and 8 µL of double-distilled water with the SYBR^®^ Green PCR Master Mix Kit (TaKaRa, Osaka, Japan) with a CFX96 Real-Time PCR Detection System (Bio-Rad, Hercules, CA, USA) using the following program: a 30 s initial denaturation at 94 °C followed by 40 cycles of denaturation at 94 °C for 5 s and annealing/extension at 61 °C for 50 s. All reactions were performed in triplicate for each sample. Bacterial 16S rDNA copy numbers are presented as the logarithm (base 10).

### 2.4. Production Performance

Laying rate and mortality were recorded daily. The body weight of the birds was recorded twice a week. Twenty eggs were randomly collected from each replicate for egg-quality measurement twice a week. All eggs were stored indoors at 18–20 °C and were measured within 4 h after laying. Egg weight was measured using an electronic scale with an accuracy of 0.01 g. Shell strength was measured with an eggshell force gauge (RH-DQ200, Runhu Instrument Co., Ltd., Guangzhou, China). Haugh units (HUs) were measured using an electronic egg tester (EMT-7300, Sanly Chemical Food Co., Ltd., Shenzhen, China).

### 2.5. Measurements of Serum Biochemical Parameters

Sixty birds from each group (12 for each replicate) were randomly selected for blood sampling twice a week. A 4-mL blood sample was collected from the wing vein of the chickens into 2 heparinised tubes (2 mL in each tube). The time between securing the bird and obtaining the blood sample did not exceed 90 s. Samples were placed in an ice bath immediately after collection and then transported to the laboratory for processing. Blood serum was separated by centrifugation for 10 min (3000× *g*) at 4 °C and stored at −20 °C until analysis. The collected serum was assayed to detect the levels of total cholesterol (T-CH), triglyceride (TG), malondialdehyde (MDA) and corticosterone (CORT) and the activities of glutathione peroxidase (GSH-Px), superoxide dismutase (SOD) and creatine kinase (CK). The concentrations of these parameters were determined by commercial analytical kits (Sigma, Thermo Fisher Scientific, Shanghai, China) with an autoanalyser (Hitachi Ltd., Tokyo, Japan).

### 2.6. Measurements of Intestinal Morphology

At 40 weeks of age, four birds per replicate in each experimental group were randomly selected for intestinal morphological observation. One-centimetre sections from the duodenum, jejunum, ileum and caecum were excised and preserved in 10% neutral buffered formalin solution. Segments were then embedded in paraffin wax, fixed onto slides and stained with hematoxylin and eosin. The observation for stained slides was performed by a Motic BA210, and the villus height (VH) and crypt depth (CD) were measured using imaging software (Motic Image Plus 2.0^ML^ Soft, Motic China Group Co., Ltd., Xiamen, China). The VCR was calculated by the ratio of villus height to crypt depth.

### 2.7. Statistical Analysis

Performance data were subjected to repeated-measures analysis, with each replicate representing an experimental unit. The parameters were averaged for each replicate. Prior to analysis, the normality of the data was verified using the Kolmogorov–Smirnov test, and the homogeneity of variance was examined by Levene’s test. Data were subjected to analysis of variance (ANOVA) using the general linear model (GLM) command in SAS version 9.3 statistical software (SAS Institute Inc., Cary, NC, USA). Statistical analyses were performed by Student’s *t*-test. All data are expressed as means ± standard deviations (SDs). Differences were considered statistically significant at *p* < 0.05.

## 3. Results

### 3.1. Indoor Airborne Parameters

**Figure 1:**
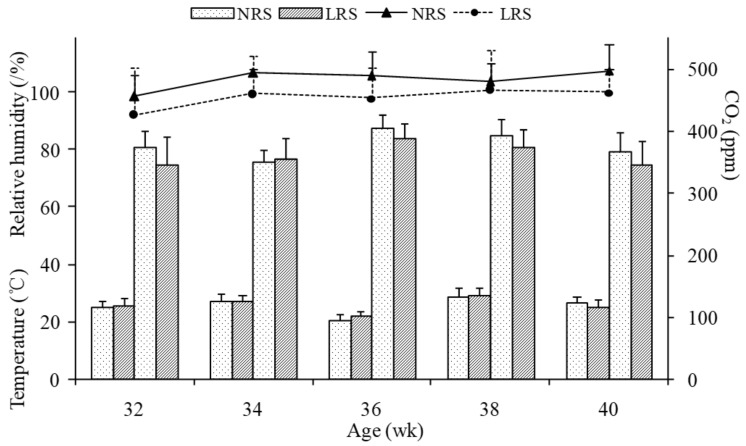
Temperature, relative humidity and CO_2_ concentration in the two housing system houses. Dotted lines represent the floor-litter housing system (LRS), and solid lines represent the plastic-net housing system (NRS). Lines connected above the bars indicate the standard deviation. wk, week.

**Figure 2:**
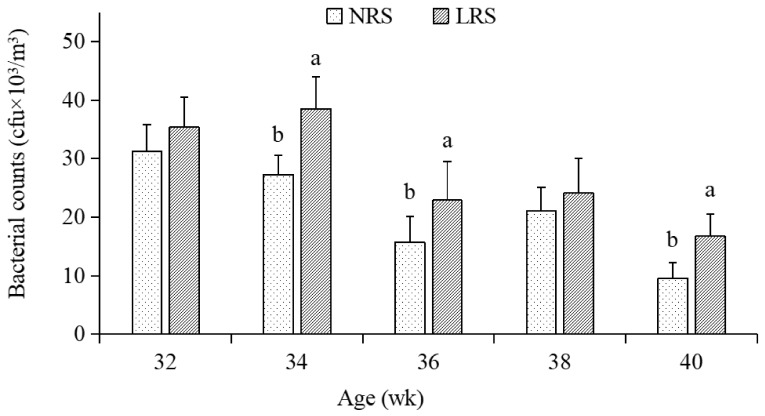
The bacterial counts of indoor aerosols in the two housing system houses. NRS, plastic-net housing system; LRS, floor-litter housing system; ^a,b^. Means with different superscripts within each period are significantly different (*p* < 0.05). Lines connected above the bars indicate the standard deviation. wk, week.

The temperature, relative humidity and CO_2_ concentration are shown in Figure 1. There were no significant differences in temperature and relative humidity during the whole period between the two housing systems (*p* > 0.05), and the CO_2_ concentration was slightly higher in the NRS than in the LRS. The indoor airborne bacteria number is shown in Figure 2. The aerosols bacterial counts in the LRS were significantly higher than that in the NRS (*p* < 0.05) during most of the experimental period (except at 32 and 38 weeks).

### 3.2. Indoor Ground Contamination

**Figure 3:**
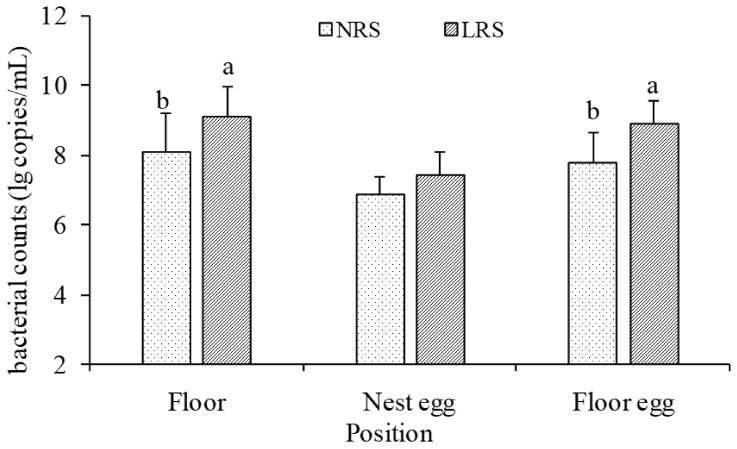
The bacterial counts on the surfaces of the floor, nest eggs and floor eggs in the two housing system houses. NRS, plastic-net housing system; LRS, floor-litter housing system; ^a,b^. Means with different superscripts within each position are significantly different (*p* < 0.05). Lines connected above the bars indicate the standard deviation.

The results of the bacterial counts (i.e., bacterial 16S rDNA copy numbers) for the surfaces of the floor and eggs are shown in Figure 3. The mean values of the bacterial counts for the floor, nest eggs and floor eggs were 8.11 ± 1.08, 6.86 ± 0.51 and 7.77 ± 0.87 units (lg copies/mL), respectively, in the NRS and 9.12 ± 0.85, 7.42 ± 0.68 and 8.91 ± 0.65 units, respectively, in the LRS. By comparing the bacterial counts from the floor and the surfaces of floor eggs, it was found that the mean values in the LRS were nearly 10 times higher than those in the NRS (*p* < 0.05).

### 3.3. Production Performance

**Figure 4:**
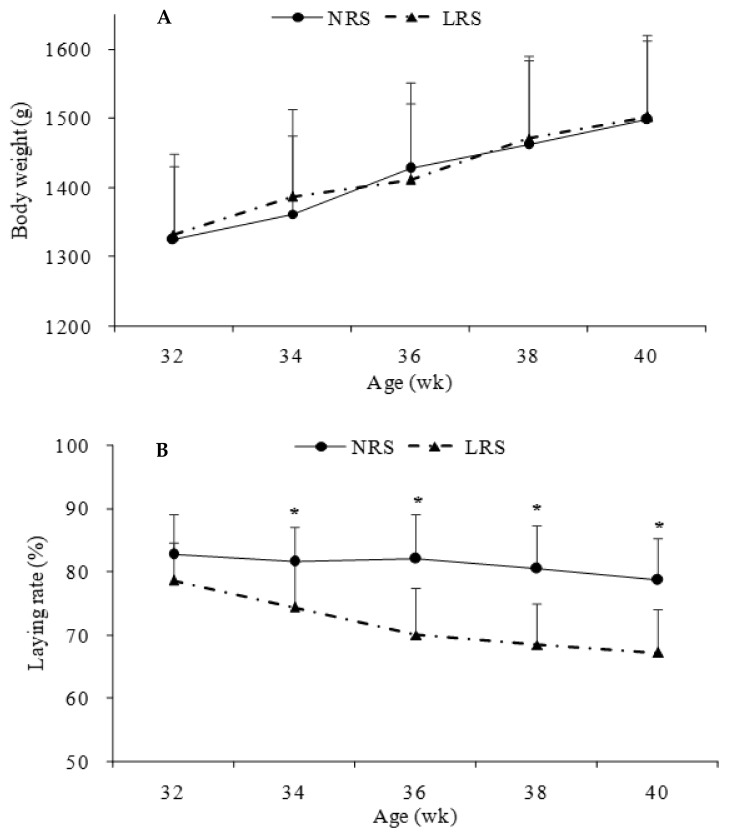
Body weights (**A**) and laying rates (**B**) of hens in the two housing system houses from 32 to 40 weeks of age. Dotted lines represent the floor-litter housing system (LRS), and solid lines represent the plastic-net housing system (NRS). ^*^ Means with asterisk superscripts within each period are significantly different (*p* < 0.05).

Production performance was measured by body weight (Figure 4A), laying rate (Figure 4B), mortality and some egg-quality traits (Table 1). No significant differences in body weight or mortality rate were found between the two groups (*p* > 0.05). However, the laying rate in the NRS group decreased gradually (by 4.14%) with age, while there was a sharp decline (from 32 to 38 weeks of age) in the LRS group (by 11.39%); the laying rate of bird in the NRS was significantly higher than that of birds in the LRS from 34 to 40 weeks of age (*p* < 0.05). The egg weight and shell strength in the NRS group were slightly higher than those in the LRS group, while the HUs were slightly lower.

### 3.4. Serum Parameters

The serum parameters of hens are shown in Table 2. Serum T-CH, TG and CORT levels were slightly lower in the NRS group than in the LRS group (*p* > 0.05), while the level of MDA in the NRS group was significantly lower than that in the LRS group (*p* < 0.05). Birds raised in the NRS had significantly higher serum concentrations of GSH-Px and SOD than those raised in the LRS (*p* < 0.05). No significant difference in serum CK concentration was found (*p* > 0.05).

### 3.5. Intestinal Morphology

The effects of housing type on the morphological parameters of the intestine are shown in Table 3. Compared to those in the LRS group, the VH and VCR in the jejunum and ileum in the NRS group were significantly increased (*p* < 0.05). Similarly, a higher VH in the duodenum and a higher VCR in the caecum were observed in the NRS group than in the LRS group (*p* < 0.05). No significant difference in the VH in the caecum or the intestinal CD was observed between the two groups (*p* > 0.05).

## 4. Discussion

Environmental conditions in poultry houses are very important to bird health. The CO_2_ concentration was observed to be higher in the environment containing wood shavings than in the environment with plastic floors by Almeida et al., (2017) [5], which was likely to be caused by the microbial degradation of organic matter accumulated in the wood shavings. In contrast, the CO_2_ concentration was slightly higher in the NRS than in the LRS during the whole period in the present study, which may be due to aerobic fermentation of the excreta from hens and the moisture contained in the excreta [11]. The similar temperatures and relative humidity values in the two housing houses were related to their identical house structure and close location.

Bioaerosols in poultry houses can cause respiratory problems due to infection as well as general respiratory stress due to constant contact with non-pathogenic bacteria [12]. The airborne bacterial content was found to be higher in LRS compared to NRS, which was similar to those obtained by Madelin et al., (1989) [13], who found that the respirable dust concentrations and numbers of airborne microorganisms were significantly higher in deep-litter systems than in net-floor systems. Litter, e.g., wood shavings, straw and rice husk, contributed directly to the airborne dust in the LRS house and are assumed to have served as a reservoir for microorganisms [14]. Birds scratching and moving the litter release particles contaminated with microorganisms into the air, where they could disperse and redeposite on the floor and eggs. This is possible to be an important cause for higher bacterial counts from the surfaces of the floor and floor eggs in LRS as compared with NRS. Similarly, Almeida et al., (2017) [5] concluded that the presence of a plastic-net floor improved plumage hygiene, as the birds had less contact with faeces. Akpobome and Fanguy (1992) [15] also observed better results with the cleaning of the broiler feathers for poultry reared on plastic floors than for those reared on wood shavings. The present study investigated higher environmental microbial pollution in LRS housing than in NRS housing, and this environmental pollution caused by poultry litter (wood shavings) may contaminate birds body and threaten their health, and consequently be a violation of animal welfare. 

The housing system influenced the performance characteristics of the hens. No significant effect of the housing system on the body weight of laying hens was observed in the present study; this was inconsistent with the results of Almeida et al., (2017) [5], who found that broilers reared on plastic nets gained more body weight than those reared on wood shavings. Moreover, Wang et al., (2015) [8] found that the growth performance of broiler chicks reared in the NRS was better than that of broiler chicks reared in the LRS. However, a novel housing system effect identified in this study was that hens reared in the NRS had a significantly higher laying rate than those reared in the LRS during 34 to 40 weeks of age, resulting in better production performance. Lower egg production in the LRS indicated negative laying performance, which may have been caused by the fact that the hens were not adapted to the LRS after being raised in cages; therefore, there was a drop in egg production. In addition, some eggs were laid in the litter and were not easily counted [16]. The different findings among studies could be attributed to differences in chicken breeds and environmental conditions. There was no difference in egg weight, shell strength or HUs between the two housing systems, similar to the results of Shimmura et al., (2010) [17], who found no significant effects of various housing systems on egg weight, egg mass or HUs in hens. In contrast, Englmaierová et al., (2014) [16] found that all of the internal and external egg quality characteristics were influenced by the housing system (litter system versus aviary).

Serum biochemical parameters are considered to be important indicators of the physiological and metabolic status of birds and are influenced by numerous factors, among which the housing system is one of the most important [8]. A previous study reported that birds raised in a conventional cage housing system had higher levels of TG and T-CH than those raised in a free-range system [18]. In the present study, the serum T-CH and TG levels in hens showed no significant differences between housing systems but were slightly higher in the LRS groups, which partially aligned with the results of Sun et al., (2015) [19], who found that birds raised in the LRS exhibited higher serum TG levels than those raised in the NRS. This may also be associated with the higher serum CK levels in the NRS group than in the LRS group, as the release of CK is thought to be proportional to the intensity and duration of exercise [20], while exercise can briefly lower serum TG and cholesterol [21]. GSH-Px and SOD are usually considered antioxidant indices that reflect the antioxidant status of animals, while MDA is the main final product of lipid peroxidation and has often been used for determining oxidative damage [22,23]. The NRS group had lower serum concentrations of MDA and higher activities of GSH-Px and SOD than those in the LRS group, which showed the superiority of the NRS in oxidation resistance, indicating a better welfare state of hens physiology. The enhanced antioxidant status induced by the NRS is likely due to lower rates of bacterial contamination of the air and ground as well as better ground hygiene and plumage conditions [10]. Similarly, the lower serum CORT concentration in the NRS group also demonstrated less physiological stress, as CORT has been suggested to be a sensitive indicator of environmental stress [24].

The health and morphology of the intestinal tract of birds are easily affected by the environment and environmental conditions [25]. The present study measured intestinal morphological parameters such as VH, CD and VCR, as they are frequently used as indicators of nutrient absorption and growth performance in hens [26]. A larger VH and VCR in the small intestine as well as a larger VCR in the caecum were observed in the NRS group. These histomorphological findings were similar to the results of Li et al., (2016) [7], who found that the VCR in the jejunum decreased at 28 days and that the VCR in the ileum decreased at 42 days in the LRS group compared with the NRS group. The worse intestinal morphology in the LRS group might be accordingly related to the higher rate of environmental bacterial contamination and microbial fermentation in faeces and litter, which could be detrimental to the growth and the repair of the intestinal mucosa, and indirectly decrease the villus height.

## 5. Conclusions

In conclusion, compared to the LRS, the NRS produced a higher-quality environment, i.e., it reduced bacterial contamination in the air and on the floor and was associated with superior laying performance and antioxidative status in hens. This system may have some positive effects on intestinal health, as indicated by a higher VH and VCR in the small intestine and higher VCR in the caecum. The current findings support the advantages of the NRS for the health and welfare of Shendan chickens between 32 and 40 weeks of age. Further studies are needed to investigate the effects of non-cage housing systems on more production traits in hens during different laying periods.

## Figures and Tables

**Table 1:** Effects of two housing systems on the production performance of hens from 32 to 40 weeks of age.

Housing System ^1^	Body Weight, g	Laying Rate, %	Mortality, %	Egg Weight, g	Eggshell Strength, kg/cm^2^	Haugh Unit
NRS	1415.04 ± 143.84	81.69 ± 8.44 ^a^	0.04 ± 0.01	46.90 ± 1.46	4.43 ± 1.01	78.70 ± 9.01
LRS	1420.76 ± 132.37	71.74 ± 8.81 ^b^	0.03 ± 0.01	46.15 ± 1.58	4.08 ± 1.13	80.07 ± 9.46

^1^ NRS, plastic-net housing system; LRS, floor-litter housing system; ^a,b^. Means with different superscripts within each column are significantly different (*p* < 0.05).

**Table 2:** Effects of two housing systems on blood serum parameters of hens from 32 to 40 weeks of age.

Housing System ^1^	T-CH, mmol/L	TG, mmol/L	MDA, ng/mL	CORT, ng/mL	GSH-Px, ng/mL	SOD, ng/mL	CK, ng/mL
NRS	4.01 ± 0.62	5.48 ± 0.70	48.02 ± 3.22 ^b^	106.73 ± 10.36	24.84 ± 3.95 ^a^	48.25 ± 6.25 ^a^	272.81 ± 21.50
LRS	4.19 ± 0.56	6.03 ± 0.77	54.28 ± 4.31 ^a^	113.45 ± 11.39	16.72 ± 2.81 ^b^	35.73 ± 5.07 ^b^	259.60 ± 25.39

^1^ NRS, plastic-net housing system; LRS, floor-litter housing system; ^a,b^.Means with different superscripts within each column are significantly different (*p* < 0.05).

**Table 3:** Effects of two housing systems on morphological parameters of the intestine in hens from 32 to 40 weeks of age.

Intestinal Parts	Item ^1^	Housing System ^2^	
		NRS	LRS
Duodenum	VH	1203.82 ± 157.49 ^a^	955.45 ± 164.07 ^b^
	CD	129.13 ± 14.92	111.61 ± 18.18
	VCR	9.29 ± 1.85	8.55 ± 1.63
Jejunum	VH	1016.03 ± 148.67 ^a^	796.52 ± 122.01 ^b^
	CD	96.94 ± 10.21	97.03 ± 10.89
	VCR	11.05 ± 1.14 ^a^	8.61 ± 1.55 ^b^
Ileum	VH	618.88 ± 34.53 ^a^	360.35 ± 21.60 ^b^
	CD	102.03 ± 9.32	104.15 ± 9.15
	VCR	6.02 ± 0.93 ^a^	3.71 ± 0.52 ^b^
Caecum	VH	605.80 ± 41.10	572.49 ± 31.90
	CD	101.83 ± 12.57	124.51 ± 13.04
	VCR	5.95 ± 0.79 ^a^	4.57 ± 0.67 ^b^

^1^ VH, villus height; CD, crypt depth; VCR, villus height to crypt depth ratio; ^2^ NRS, plastic-net housing system; LRS, floor-litter housing system; ^a,b^. Means with different superscripts within each row are significantly different (*p* < 0.05).

## Data Availability

Not applicable.

## References

[1] Fu D.Z., Zhang D.X., Xu G.Y., Li K.Y., Wang Q., Zhang Z.B., Li J.Y., Chen Y., Jia Y.X., Qu L.J. (2015). Effects of different rearing systems on meat production traits and meat fiber microstructure of Beijing-you chicken. Anim. Sci. J..

[2] Ferrante V., Susanna L., Giuseppe V., Cavalchini L.G. (2009). Effects of two different rearing systems (organic and barn) on production performance, animal welfare traits and egg quality characteristics in laying hens. Ital. J. Anim. Sci..

[3] Tauson R. (2005). Management and housing systems for layers—Effects on welfare and production. World Poult. Sci. J..

[4] Zofia S., Magdalena D., Jadwiga T., Józefa K., Anna A. (2020). The effect of the type of non-caged housing system, genotype and age on the behaviour of laying hens. Animals.

[5] Almeida E.A., Arantes de Souza L.F., Sant A.C., Bahiense R.N., Macari M., Furlan R.L. (2017). Poultry rearing on perforated plastic floors and the effect on air quality, growth performance, and carcass injuries—Experiment 1: Thermal comfort. Poult. Sci..

[6] Zhang C., Richard H., Chen K.K., Zhao X.H., Yang L., Wang L., Chen X.Y., Jin S.H., Geng Z.Y. (2018). Effects of different rearing systems on growth performance, carcass traits, meat quality and serum biochemical parameters of Chaohu ducks. Anim. Sci. J..

[7] Li J.H., Miao Z.Q., Tian W.X., Yang Y., Wang J.D., Yang Y. (2016). Effects of different rearing systems on growth, small intestinal morphology and selected indices of fermentation status in broilers. Anim. Sci. J..

[8] Wang Y., Ru Y.J., Liu J.H., Chang W.H., Zhang S., Yan H.J., Zheng A.J., Lou R.Y., Liu Z.Y., Cai H.Y. (2015). Effects of different rearing systems on growth performance, nutrients digestibility, digestive organ weight, carcass traits, and energy utilization in male broiler chickens. Livest. Sci..

[9] Mariam E.A., Afaf Y.A., Faten K.A., Gehan R., Magdy M.M. (2012). Production performance of different broiler breeds under different housing systems. Int. J. Poult. Sci..

[10] Heerkens J.L.T., Delezie E., Kempen I., Zoons J., Ampe B., Rodenburg T.B., Tuyttens F.A.M. (2015). Specific characteristics of the aviary housing system affect plumage condition, mortality and production in laying hens. Poult. Sci..

[11] Calvet S., Estellés F., Cambra-López M., Torres A.G., Van den Weghe H.F.A. (2011). The influence of broiler activity, growth rate, and litter on carbon dioxide balances for the determination of ventilation flow rates in broiler production. Poult. Sci..

[12] Cambra-López M., Aarnink A., Zhao Y., Calvet S., Torres A. (2009). Airborne particulate matter from livestock production systems: A review of an air pollution problem. Environ. Pollut..

[13] Madelin T.M., Wathes C.M. (1989). Air hygiene in a broiler house: Comparison of deep litter with raised netting floors. Brit. Poult. Sci..

[14] Woodward C.L., Park S.Y., Jackson D.R., Li X., Birkhold S.G., Pillal S.D., Ricke S.C. (2004). Optimization and comparison of bacterial load and sampling time for bioaerosol detection systems in a poultry layer house. J. Appl. Poult. Res..

[15] Akpobome G.O., Fanguy R.C. (1992). Evaluation of cage floor systems for production of commercial broilers. Poult. Sci..

[16] Englmaierová M., Tumová E., Charvátová V., Skřivan M. (2014). Effects of laying hens housing system on laying performance, egg quality characteristics, and egg microbial contamination. Czech J. Anim. Sci..

[17] Shimmura T., Hirahara S., Azuma T., Suzuki T., Eguchi Y., Uetake K., Tanaka T. (2010). Multi-factorial investigation of various housing systems for laying hens. Br. Poult. Sci..

[18] Jin S.H., Fan X.F., Yang L., He T.T., Xu Y., Chen X.Y., Liu P., Geng Z.Y. (2019). Effects of rearing systems on growth performance, carcass yield, meat quality, lymphoid organ indices, and serum biochemistry of Wannan Yellow chickens. Anim. Sci. J..

[19] Sun L., Wang Y., Xie H. (2015). Effects of feeding modes and low nutrient levels on growth performance and serum biochemical indexes of meat geese aged from 5 to 8 weeks. Chin. J. Anim. Nutr..

[20] Apple F.S. (1981). Presence of creatine kinase mb isoenzyme during marathon training. N. Engl. J. Med..

[21] Oscai L., Patterson J., Bogard D., Beck R., Rothermel B. (1972). Normalization of serum triglyceride and lipoprotein electrophoretic patterns by exercise. Am. J. Cardiol..

[22] Schulz J.B., Lindenau J., Seyfried J., Dichgans J. (2000). Glutathione, oxidative stress and neurodegeneration. Eur. J. Biochem..

[23] Wu B., Cui H., Xi P., Jing F., Huang J. (2013). Investigation of the serum oxidative stress in broilers fed on diets supplemented with nickel chloride. Health.

[24] Olanrewaju H.A., Wongpichet S., Thaxton J.P., Dozier W.A., Branton S.L. (2006). Stress and acid-base balance in chickens. Poult. Sci..

[25] Camp J., Kanther M., Semova I., Rawls J. (2009). Patterns and scales in gastrointestinal microbial ecology. Gastroenterology.

[26] Ruangpanit Y., Matsushita K., Mukai K., Kikusato M. (2020). Effect of trehalose supplementation on growth performance and intestinal morphology in broiler chickens. Vet. Anim. Sci..

